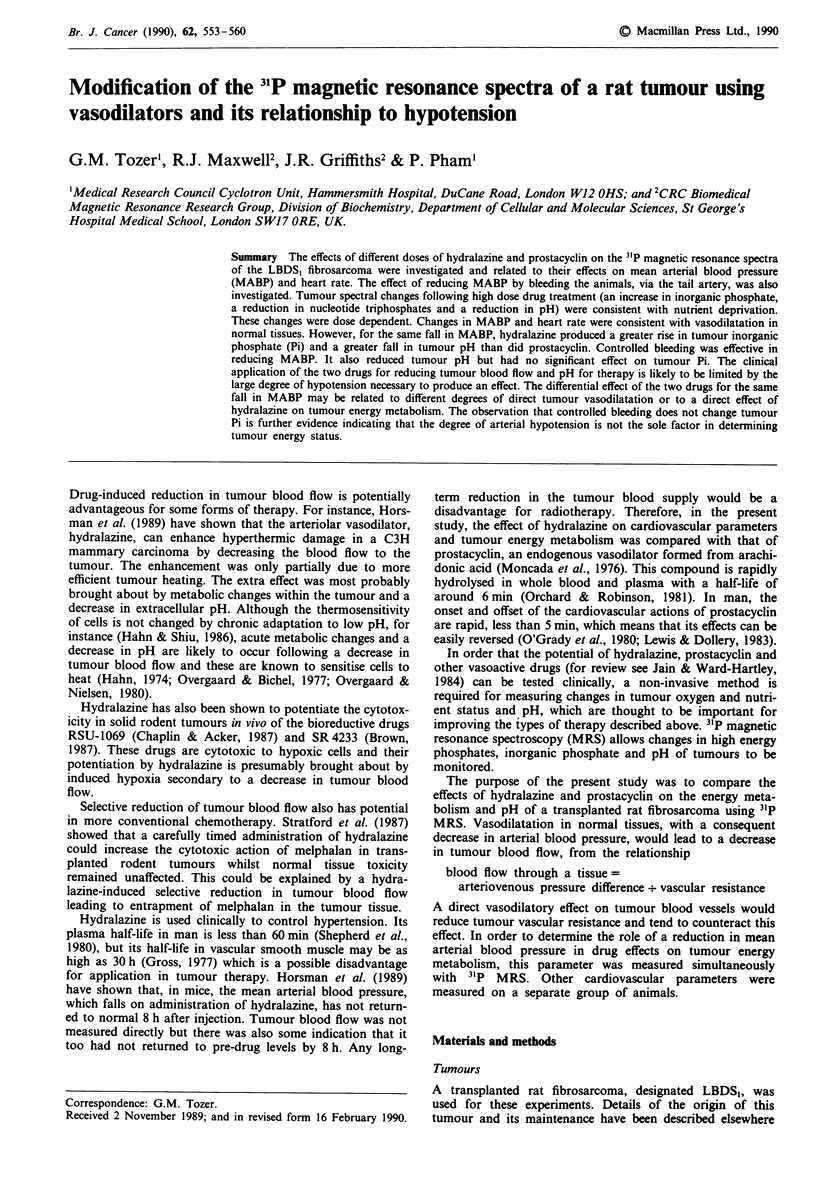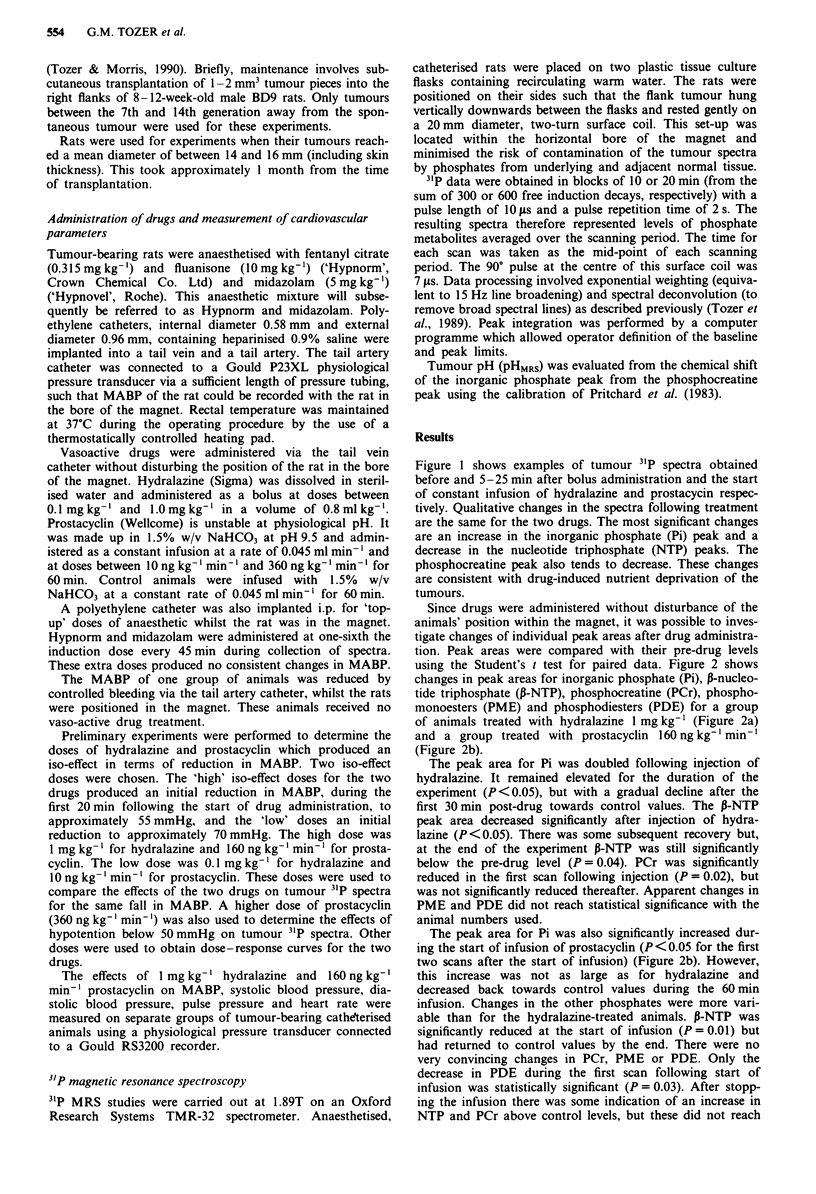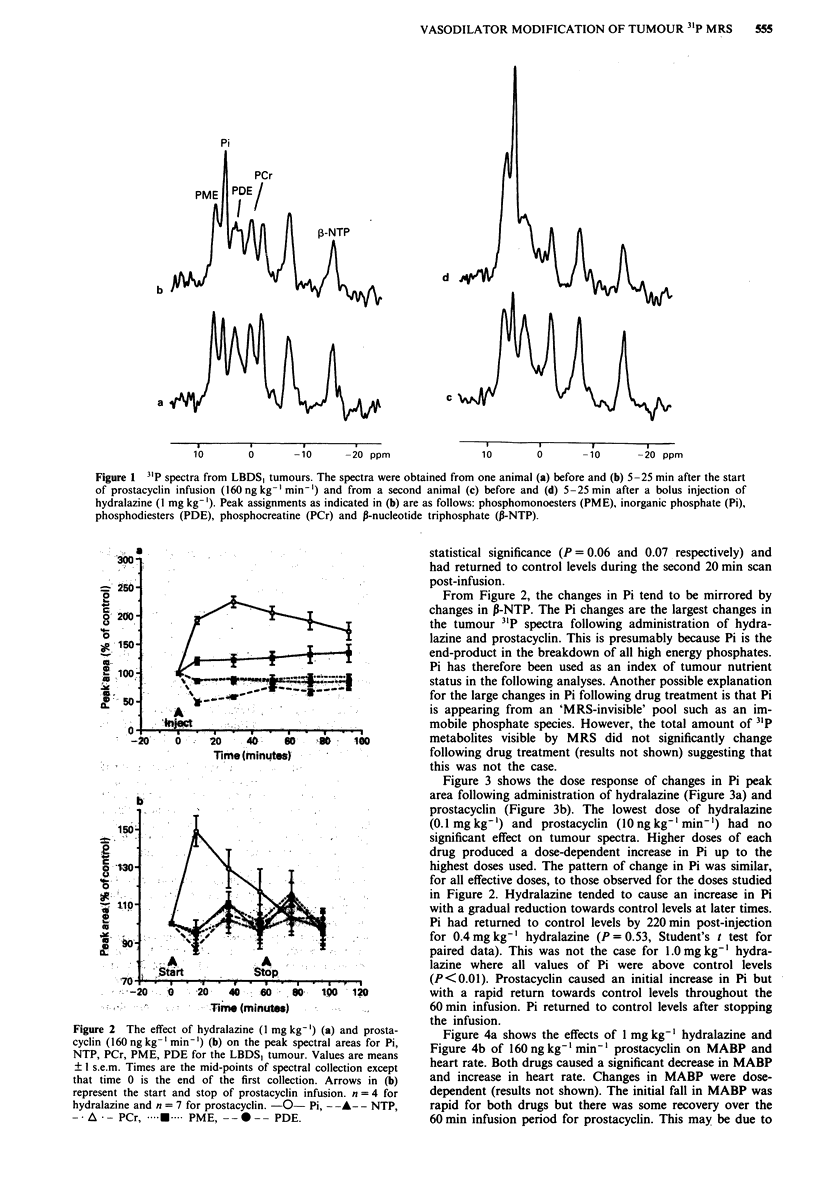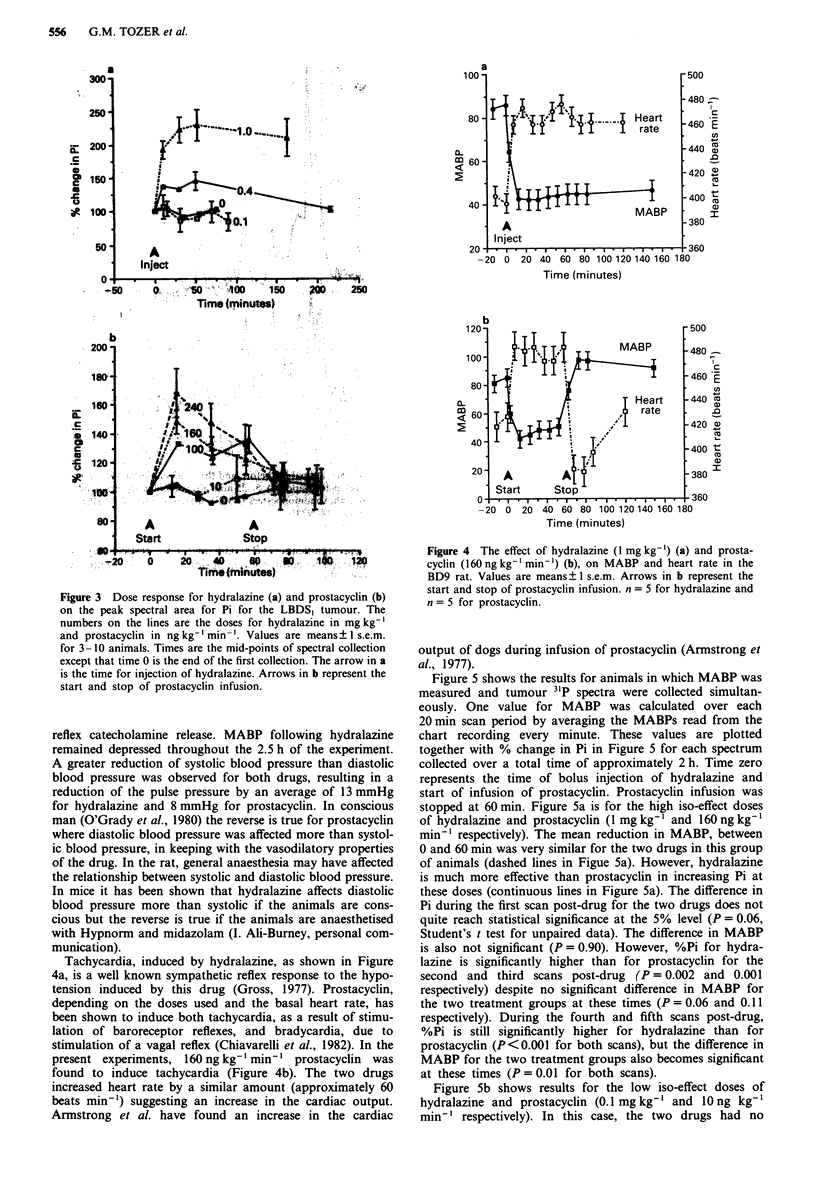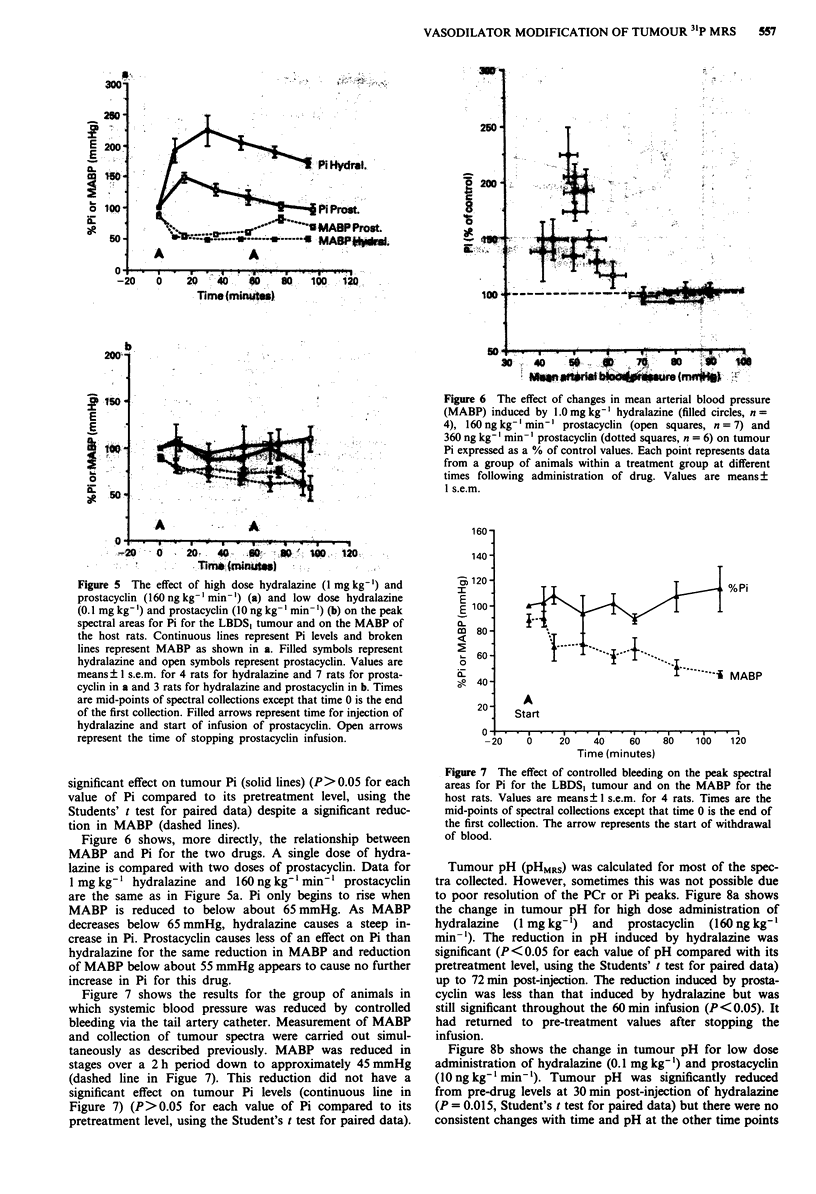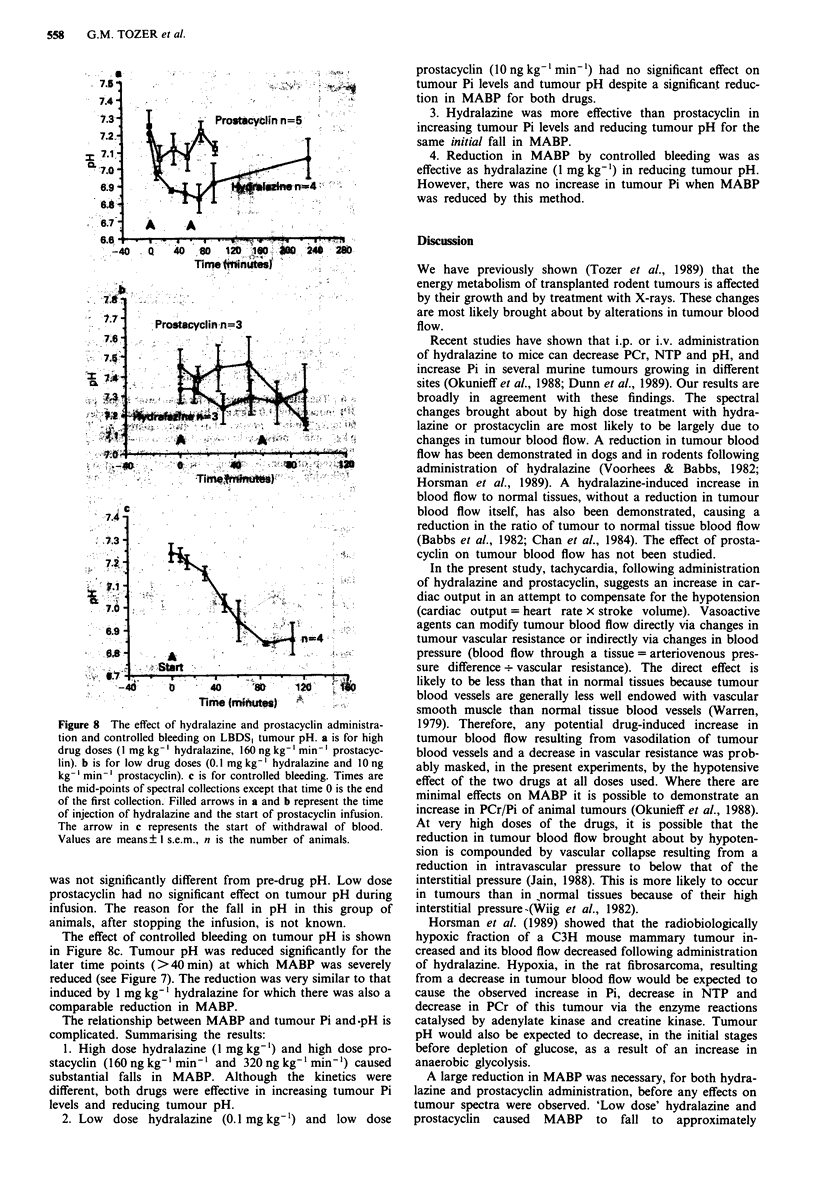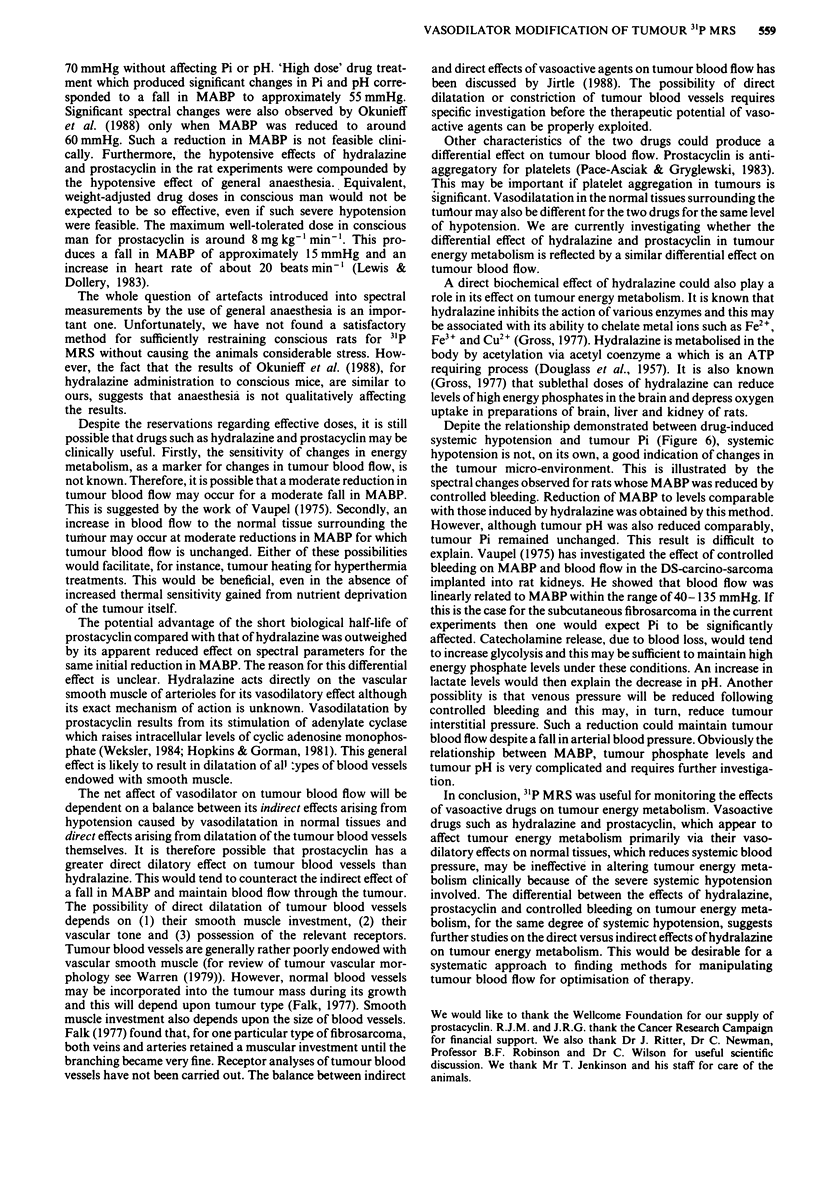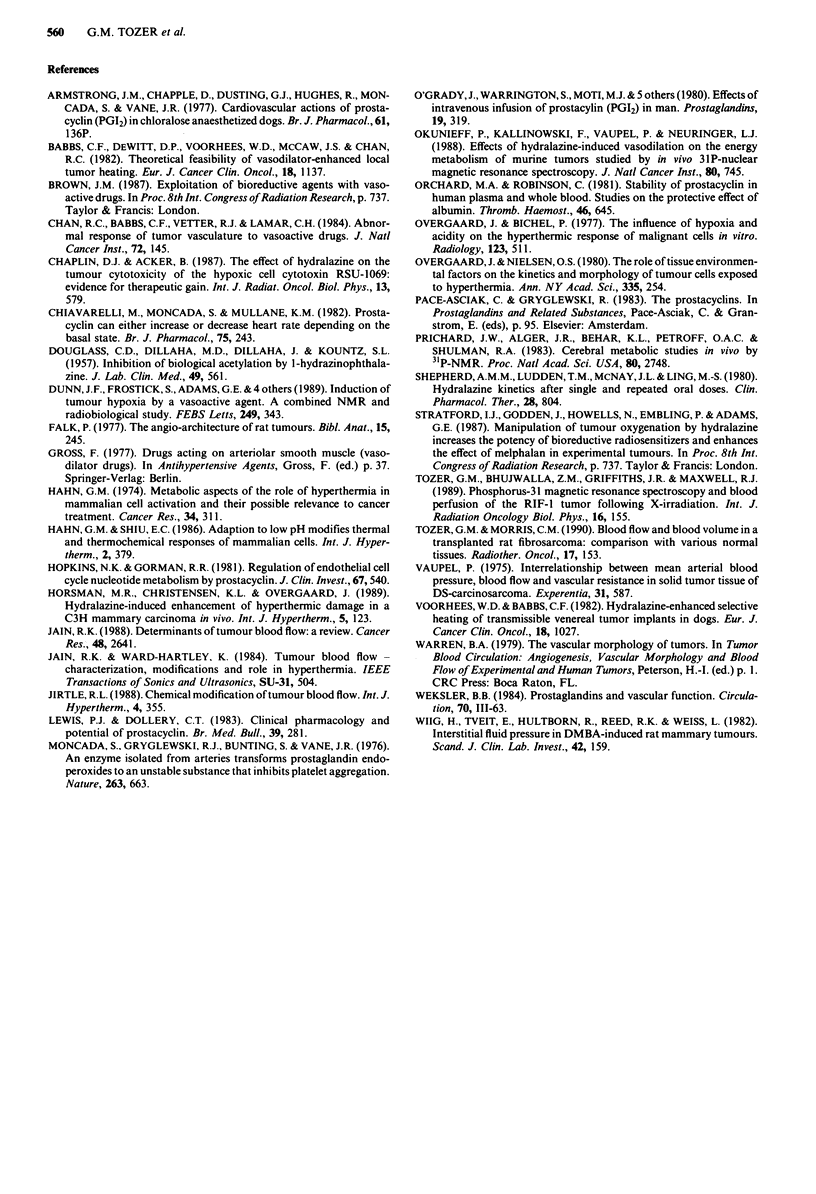# Modification of the 31P magnetic resonance spectra of a rat tumour using vasodilators and its relationship to hypotension.

**DOI:** 10.1038/bjc.1990.329

**Published:** 1990-10

**Authors:** G. M. Tozer, R. J. Maxwell, J. R. Griffiths, P. Pham

**Affiliations:** Medical Research Council Cyclotron Unit, Hammersmith Hospital, London, UK.

## Abstract

The effects of different doses of hydralazine and prostacyclin on the 31P magnetic resonance spectra of the LBDS1 fibrosarcoma were investigated and related to their effects on mean arterial blood pressure (MABP) and heart rate. The effect of reducing MABP by bleeding the animals, via the tail artery, was also investigated. Tumour spectral changes following high dose drug treatment (an increase in inorganic phosphate, a reduction in nucleotide triphosphates and a reduction in pH) were consistent with nutrient deprivation. These changes were dose dependent. Changes in MABP and heart rate were consistent with vasodilatation in normal tissues. However, for the same fall in MABP, hydralazine produced a greater rise in tumour inorganic phosphate (Pi) and a greater fall in tumour pH than did prostacyclin. Controlled bleeding was effective in reducing MABP. It also reduced tumour pH but had no significant effect on tumour Pi. The clinical application of the two drugs for reducing tumour blood flow and pH for therapy is likely to be limited by the large degree of hypotension necessary to produce an effect. The differential effect of the two drugs for the same fall in MABP may be related to different degrees of direct tumour vasodilatation or to a direct effect of hydralazine on tumour energy metabolism. The observation that controlled bleeding does not change tumour Pi is further evidence indicating that the degree of arterial hypotension is not the sole factor in determining tumour energy status.


Br. J. Cancer (1990), 62, 553-560 © Macmillan Press Ltd., 1990

### Modification of the $^{31}$P magnetic resonance spectra of a rat tumour using vasodilators and its relationship to hypotension

G.M. Tozer[1], R.J. Maxwell[2], J.R. Griffiths[2] & P. Pham[1]

[1]*Medical Research Council Cyclotron Unit, Hammersmith Hospital, DuCane Road, London W12 0HS; and* [2]*CRC Biomedical Magnetic Resonance Research Group, Division of Biochemistry, Department of Cellular and Molecular Sciences, St George's Hospital Medical School, London SW17 0RE, UK.*

**Summary** The effects of different doses of hydralazine and prostacyclin on the $^{31}$P magnetic resonance spectra of the $LBDS_1$ fibrosarcoma were investigated and related to their effects on mean arterial blood pressure (MABP) and heart rate. The effect of reducing MABP by bleeding the animals, via the tail artery, was also investigated. Tumour spectral changes following high dose drug treatment (an increase in inorganic phosphate, a reduction in nucleotide triphosphates and a reduction in pH) were consistent with nutrient deprivation. These changes were dose dependent. Changes in MABP and heart rate were consistent with vasodilatation in normal tissues. However, for the same fall in MABP, hydralazine produced a greater rise in tumour inorganic phosphate (Pi) and a greater fall in tumour pH than did prostacyclin. Controlled bleeding was effective in reducing MABP. It also reduced tumour pH but had no significant effect on tumour Pi. The clinical application of the two drugs for reducing tumour blood flow and pH for therapy is likely to be limited by the large degree of hypotension necessary to produce an effect. The differential effect of the two drugs for the same fall in MABP may be related to different degrees of direct tumour vasodilatation or to a direct effect of hydralazine on tumour energy metabolism. The observation that controlled bleeding does not change tumour Pi is further evidence indicating that the degree of arterial hypotension is not the sole factor in determining tumour energy status.

Drug-induced reduction in tumour blood flow is potentially advantageous for some forms of therapy. For instance, Horsman *et al.* (1989) have shown that the arteriolar vasodilator, hydralazine, can enhance hyperthermic damage in a C3H mammary carcinoma by decreasing the blood flow to the tumour. The enhancement was only partially due to more efficient tumour heating. The extra effect was most probably brought about by metabolic changes within the tumour and a decrease in extracellular pH. Although the thermosensitivity of cells is not changed by chronic adaptation to low pH, for instance (Hahn & Shiu, 1986), acute metabolic changes and a decrease in pH are likely to occur following a decrease in tumour blood flow and these are known to sensitise cells to heat (Hahn, 1974; Overgaard & Bichel, 1977; Overgaard & Nielsen, 1980).

Hydralazine has also been shown to potentiate the cytotoxicity in solid rodent tumours *in vivo* of the bioreductive drugs RSU-1069 (Chaplin & Acker, 1987) and SR 4233 (Brown, 1987). These drugs are cytotoxic to hypoxic cells and their potentiation by hydralazine is presumably brought about by induced hypoxia secondary to a decrease in tumour blood flow.

Selective reduction of tumour blood flow also has potential in more conventional chemotherapy. Stratford *et al.* (1987) showed that a carefully timed administration of hydralazine could increase the cytotoxic action of melphalan in transplanted rodent tumours whilst normal tissue toxicity remained unaffected. This could be explained by a hydralazine-induced selective reduction in tumour blood flow leading to entrapment of melphalan in the tumour tissue.

Hydralazine is used clinically to control hypertension. Its plasma half-life in man is less than 60 min (Shepherd *et al.*, 1980), but its half-life in vascular smooth muscle may be as high as 30 h (Gross, 1977) which is a possible disadvantage for application in tumour therapy. Horsman *et al.* (1989) have shown that, in mice, the mean arterial blood pressure, which falls on administration of hydralazine, has not returned to normal 8 h after injection. Tumour blood flow was not measured directly but there was also some indication that it too had not returned to pre-drug levels by 8 h. Any long-term reduction in the tumour blood supply would be a disadvantage for radiotherapy. Therefore, in the present study, the effect of hydralazine on cardiovascular parameters and tumour energy metabolism was compared with that of prostacyclin, an endogenous vasodilator formed from arachidonic acid (Moncada *et al.*, 1976). This compound is rapidly hydrolysed in whole blood and plasma with a half-life of around 6 min (Orchard & Robinson, 1981). In man, the onset and offset of the cardiovascular actions of prostacyclin are rapid, less than 5 min, which means that its effects can be easily reversed (O'Grady *et al.*, 1980; Lewis & Dollery, 1983).

In order that the potential of hydralazine, prostacyclin and other vasoactive drugs (for review see Jain & Ward-Hartley, 1984) can be tested clinically, a non-invasive method is required for measuring changes in tumour oxygen and nutrient status and pH, which are thought to be important for improving the types of therapy described above. $^{31}$P magnetic resonance spectroscopy (MRS) allows changes in high energy phosphates, inorganic phosphate and pH of tumours to be monitored.

The purpose of the present study was to compare the effects of hydralazine and prostacyclin on the energy metabolism and pH of a transplanted rat fibrosarcoma using $^{31}$P MRS. Vasodilatation in normal tissues, with a consequent decrease in arterial blood pressure, would lead to a decrease in tumour blood flow, from the relationship

$$\text{blood flow through a tissue} = \text{arteriovenous pressure difference} \div \text{vascular resistance}$$

A direct vasodilatory effect on tumour blood vessels would reduce tumour vascular resistance and tend to counteract this effect. In order to determine the role of a reduction in mean arterial blood pressure in drug effects on tumour energy metabolism, this parameter was measured simultaneously with $^{31}$P MRS. Other cardiovascular parameters were measured on a separate group of animals.

### Materials and methods

### *Tumours*

A transplanted rat fibrosarcoma, designated $LBDS_1$, was used for these experiments. Details of the origin of this tumour and its maintenance have been described elsewhere

Correspondence: G.M. Tozer.
Received 2 November 1989; and in revised form 16 February 1990.

(Tozer & Morris, 1990). Briefly, maintenance involves subcutaneous transplantation of 1–2 $mm^3$ tumour pieces into the right flanks of 8–12-week-old male BD9 rats. Only tumours between the 7th and 14th generation away from the spontaneous tumour were used for these experiments.

Rats were used for experiments when their tumours reached a mean diameter of between 14 and 16 mm (including skin thickness). This took approximately 1 month from the time of transplantation.

*Administration of drugs and measurement of cardiovascular parameters*

Tumour-bearing rats were anaesthetised with fentanyl citrate (0.315 mg $kg^{-1}$) and fluanisone (10 mg $kg^{-1}$) ('Hypnorm', Crown Chemical Co. Ltd) and midazolam (5 mg $kg^{-1}$) ('Hypnovel', Roche). This anaesthetic mixture will subsequently be referred to as Hypnorm and midazolam. Polyethylene catheters, internal diameter 0.58 mm and external diameter 0.96 mm, containing heparinised 0.9% saline were implanted into a tail vein and a tail artery. The tail artery catheter was connected to a Gould P23XL physiological pressure transducer via a sufficient length of pressure tubing, such that MABP of the rat could be recorded with the rat in the bore of the magnet. Rectal temperature was maintained at 37°C during the operating procedure by the use of a thermostatically controlled heating pad.

Vasoactive drugs were administered via the tail vein catheter without disturbing the position of the rat in the bore of the magnet. Hydralazine (Sigma) was dissolved in sterilised water and administered as a bolus at doses between 0.1 mg $kg^{-1}$ and 1.0 mg $kg^{-1}$ in a volume of 0.8 ml $kg^{-1}$. Prostacyclin (Wellcome) is unstable at physiological pH. It was made up in 1.5% w/v $NaHCO_3$ at pH 9.5 and administered as a constant infusion at a rate of 0.045 ml $min^{-1}$ and at doses between 10 ng $kg^{-1}$ $min^{-1}$ and 360 ng $kg^{-1}$ $min^{-1}$ for 60 min. Control animals were infused with 1.5% w/v $NaHCO_3$ at a constant rate of 0.045 ml $min^{-1}$ for 60 min.

A polyethylene catheter was also implanted i.p. for 'top-up' doses of anaesthetic whilst the rat was in the magnet. Hypnorm and midazolam were administered at one-sixth the induction dose every 45 min during collection of spectra. These extra doses produced no consistent changes in MABP.

The MABP of one group of animals was reduced by controlled bleeding via the tail artery catheter, whilst the rats were positioned in the magnet. These animals received no vaso-active drug treatment.

Preliminary experiments were performed to determine the doses of hydralazine and prostacyclin which produced an iso-effect in terms of reduction in MABP. Two iso-effect doses were chosen. The 'high' iso-effect doses for the two drugs produced an initial reduction in MABP, during the first 20 min following the start of drug administration, to approximately 55 mmHg, and the 'low' doses an initial reduction to approximately 70 mmHg. The high dose was 1 mg $kg^{-1}$ for hydralazine and 160 ng $kg^{-1}$ $min^{-1}$ for prostacyclin. The low dose was 0.1 mg $kg^{-1}$ for hydralazine and 10 ng $kg^{-1}$ $min^{-1}$ for prostacyclin. These doses were used to compare the effects of the two drugs on tumour $^{31}P$ spectra for the same fall in MABP. A higher dose of prostacyclin (360 ng $kg^{-1}$ $min^{-1}$) was also used to determine the effects of hypotention below 50 mmHg on tumour $^{31}P$ spectra. Other doses were used to obtain dose–response curves for the two drugs.

The effects of 1 mg $kg^{-1}$ hydralazine and 160 ng $kg^{-1}$ $min^{-1}$ prostacyclin on MABP, systolic blood pressure, diastolic blood pressure, pulse pressure and heart rate were measured on separate groups of tumour-bearing catheterised animals using a physiological pressure transducer connected to a Gould RS3200 recorder.

*$^{31}P$ magnetic resonance spectroscopy*

$^{31}P$ MRS studies were carried out at 1.89T on an Oxford Research Systems TMR-32 spectrometer. Anaesthetised, catheterised rats were placed on two plastic tissue culture flasks containing recirculating warm water. The rats were positioned on their sides such that the flank tumour hung vertically downwards between the flasks and rested gently on a 20 mm diameter, two-turn surface coil. This set-up was located within the horizontal bore of the magnet and minimised the risk of contamination of the tumour spectra by phosphates from underlying and adjacent normal tissue.

$^{31}P$ data were obtained in blocks of 10 or 20 min (from the sum of 300 or 600 free induction decays, respectively) with a pulse length of 10 μs and a pulse repetition time of 2 s. The resulting spectra therefore represented levels of phosphate metabolites averaged over the scanning period. The time for each scan was taken as the mid-point of each scanning period. The 90° pulse at the centre of this surface coil was 7 μs. Data processing involved exponential weighting (equivalent to 15 Hz line broadening) and spectral deconvolution (to remove broad spectral lines) as described previously (Tozer *et al.*, 1989). Peak integration was performed by a computer programme which allowed operator definition of the baseline and peak limits.

Tumour pH ($pH_{MRS}$) was evaluated from the chemical shift of the inorganic phosphate peak from the phosphocreatine peak using the calibration of Pritchard *et al.* (1983).

### Results

Figure 1 shows examples of tumour $^{31}P$ spectra obtained before and 5–25 min after bolus administration and the start of constant infusion of hydralazine and prostacyclin respectively. Qualitative changes in the spectra following treatment are the same for the two drugs. The most significant changes are an increase in the inorganic phosphate (Pi) peak and a decrease in the nucleotide triphosphate (NTP) peaks. The phosphocreatine peak also tends to decrease. These changes are consistent with drug-induced nutrient deprivation of the tumours.

Since drugs were administered without disturbance of the animals' position within the magnet, it was possible to investigate changes of individual peak areas after drug administration. Peak areas were compared with their pre-drug levels using the Student's *t* test for paired data. Figure 2 shows changes in peak areas for inorganic phosphate (Pi), β-nucleotide triphosphate (β-NTP), phosphocreatine (PCr), phosphomonoesters (PME) and phosphodiesters (PDE) for a group of animals treated with hydralazine 1 mg $kg^{-1}$ (Figure 2a) and a group treated with prostacyclin 160 ng $kg^{-1}$ $min^{-1}$ (Figure 2b).

The peak area for Pi was doubled following injection of hydralazine. It remained elevated for the duration of the experiment ($P < 0.05$), but with a gradual decline after the first 30 min post-drug towards control values. The β-NTP peak area decreased significantly after injection of hydralazine ($P < 0.05$). There was some subsequent recovery but, at the end of the experiment β-NTP was still significantly below the pre-drug level ($P = 0.04$). PCr was significantly reduced in the first scan following injection ($P = 0.02$), but was not significantly reduced thereafter. Apparent changes in PME and PDE did not reach statistical significance with the animal numbers used.

The peak area for Pi was also significantly increased during the start of infusion of prostacyclin ($P < 0.05$ for the first two scans after the start of infusion) (Figure 2b). However, this increase was not as large as for hydralazine and decreased back towards control values during the 60 min infusion. Changes in the other phosphates were more variable than for the hydralazine-treated animals. β-NTP was significantly reduced at the start of infusion ($P = 0.01$) but had returned to control values by the end. There were no very convincing changes in PCr, PME or PDE. Only the decrease in PDE during the first scan following start of infusion was statistically significant ($P = 0.03$). After stopping the infusion there was some indication of an increase in NTP and PCr above control levels, but these did not reach

**Figure 1** $^{31}$P spectra from $LBDS_1$ tumours. The spectra were obtained from one animal (**a**) before and (**b**) 5–25 min after the start of prostacyclin infusion (160 ng $kg^{-1}$ $min^{-1}$) and from a second animal (**c**) before and (**d**) 5–25 min after a bolus injection of hydralazine (1 mg $kg^{-1}$). Peak assignments as indicated in (**b**) are as follows: phosphomonoesters (PME), inorganic phosphate (Pi), phosphodiesters (PDE), phosphocreatine (PCr) and β-nucleotide triphosphate (β-NTP).

**Figure 2** The effect of hydralazine (1 mg $kg^{-1}$) (**a**) and prostacyclin (160 ng $kg^{-1}$ $min^{-1}$) (**b**) on the peak spectral areas for Pi, NTP, PCr, PME, PDE for the $LBDS_1$ tumour. Values are means ± 1 s.e.m. Times are the mid-points of spectral collection except that time 0 is the end of the first collection. Arrows in (**b**) represent the start and stop of prostacyclin infusion. $n = 4$ for hydralazine and $n = 7$ for prostacyclin. —O— Pi, - -▲- - NTP, - · △ · - PCr, ····■···· PME, - - ● - - PDE.

statistical significance ($P = 0.06$ and 0.07 respectively) and had returned to control levels during the second 20 min scan post-infusion.

From Figure 2, the changes in Pi tend to be mirrored by changes in β-NTP. The Pi changes are the largest changes in the tumour $^{31}$P spectra following administration of hydralazine and prostacyclin. This is presumably because Pi is the end-product in the breakdown of all high energy phosphates. Pi has therefore been used as an index of tumour nutrient status in the following analyses. Another possible explanation for the large changes in Pi following drug treatment is that Pi is appearing from an 'MRS-invisible' pool such as an immobile phosphate species. However, the total amount of $^{31}$P metabolites visible by MRS did not significantly change following drug treatment (results not shown) suggesting that this was not the case.

Figure 3 shows the dose response of changes in Pi peak area following administration of hydralazine (Figure 3a) and prostacyclin (Figure 3b). The lowest dose of hydralazine (0.1 mg $kg^{-1}$) and prostacyclin (10 ng $kg^{-1}$ $min^{-1}$) had no significant effect on tumour spectra. Higher doses of each drug produced a dose-dependent increase in Pi up to the highest doses used. The pattern of change in Pi was similar, for all effective doses, to those observed for the doses studied in Figure 2. Hydralazine tended to cause an increase in Pi with a gradual reduction towards control levels at later times. Pi had returned to control levels by 220 min post-injection for 0.4 mg $kg^{-1}$ hydralazine ($P = 0.53$, Student's $t$ test for paired data). This was not the case for 1.0 mg $kg^{-1}$ hydralazine where all values of Pi were above control levels ($P < 0.01$). Prostacyclin caused an initial increase in Pi but with a rapid return towards control levels throughout the 60 min infusion. Pi returned to control levels after stopping the infusion.

Figure 4a shows the effects of 1 mg $kg^{-1}$ hydralazine and Figure 4b of 160 ng $kg^{-1}$ $min^{-1}$ prostacyclin on MABP and heart rate. Both drugs caused a significant decrease in MABP and increase in heart rate. Changes in MABP were dose-dependent (results not shown). The initial fall in MABP was rapid for both drugs but there was some recovery over the 60 min infusion period for prostacyclin. This may be due to

**Figure 3** Dose response for hydralazine (**a**) and prostacyclin (**b**) on the peak spectral area for Pi for the $LBDS_1$ tumour. The numbers on the lines are the doses for hydralazine in mg $kg^{-1}$ and prostacyclin in ng $kg^{-1}$ $min^{-1}$. Values are means ± 1 s.e.m. for 3–10 animals. Times are the mid-points of spectral collection except that time 0 is the end of the first collection. The arrow in **a** is the time for injection of hydralazine. Arrows in **b** represent the start and stop of prostacyclin infusion.

**Figure 4** The effect of hydralazine (1 mg $kg^{-1}$) (**a**) and prostacyclin (160 ng $kg^{-1}$ $min^{-1}$) (**b**), on MABP and heart rate in the BD9 rat. Values are means ± 1 s.e.m. Arrows in **b** represent the start and stop of prostacyclin infusion. $n = 5$ for hydralazine and $n = 5$ for prostacyclin.

reflex catecholamine release. MABP following hydralazine remained depressed throughout the 2.5 h of the experiment. A greater reduction of systolic blood pressure than diastolic blood pressure was observed for both drugs, resulting in a reduction of the pulse pressure by an average of 13 mmHg for hydralazine and 8 mmHg for prostacyclin. In conscious man (O'Grady *et al.*, 1980) the reverse is true for prostacyclin where diastolic blood pressure was affected more than systolic blood pressure, in keeping with the vasodilatory properties of the drug. In the rat, general anaesthesia may have affected the relationship between systolic and diastolic blood pressure. In mice it has been shown that hydralazine affects diastolic blood pressure more than systolic if the animals are conscious but the reverse is true if the animals are anaesthetised with Hypnorm and midazolam (I. Ali-Burney, personal communication).

Tachycardia, induced by hydralazine, as shown in Figure 4a, is a well known sympathetic reflex response to the hypotension induced by this drug (Gross, 1977). Prostacyclin, depending on the doses used and the basal heart rate, has been shown to induce both tachycardia, as a result of stimulation of baroreceptor reflexes, and bradycardia, due to stimulation of a vagal reflex (Chiavarelli *et al.*, 1982). In the present experiments, 160 ng $kg^{-1}$ $min^{-1}$ prostacyclin was found to induce tachycardia (Figure 4b). The two drugs increased heart rate by a similar amount (approximately 60 beats $min^{-1}$) suggesting an increase in the cardiac output. Armstrong *et al.* have found an increase in the cardiac output of dogs during infusion of prostacyclin (Armstrong *et al.*, 1977).

Figure 5 shows the results for animals in which MABP was measured and tumour $^{31}P$ spectra were collected simultaneously. One value for MABP was calculated over each 20 min scan period by averaging the MABPs read from the chart recording every minute. These values are plotted together with % change in Pi in Figure 5 for each spectrum collected over a total time of approximately 2 h. Time zero represents the time of bolus injection of hydralazine and start of infusion of prostacyclin. Prostacyclin infusion was stopped at 60 min. Figure 5a is for the high iso-effect doses of hydralazine and prostacyclin (1 mg $kg^{-1}$ and 160 ng $kg^{-1}$ $min^{-1}$ respectively). The mean reduction in MABP, between 0 and 60 min was very similar for the two drugs in this group of animals (dashed lines in Figue 5a). However, hydralazine is much more effective than prostacyclin in increasing Pi at these doses (continuous lines in Figure 5a). The difference in Pi during the first scan post-drug for the two drugs does not quite reach statistical significance at the 5% level ($P = 0.06$, Student's $t$ test for unpaired data). The difference in MABP is also not significant ($P = 0.90$). However, %Pi for hydralazine is significantly higher than for prostacyclin for the second and third scans post-drug ($P = 0.002$ and 0.001 respectively) despite no significant difference in MABP for the two treatment groups at these times ($P = 0.06$ and 0.11 respectively). During the fourth and fifth scans post-drug, %Pi is still significantly higher for hydralazine than for prostacyclin ($P < 0.001$ for both scans), but the difference in MABP for the two treatment groups also becomes significant at these times ($P = 0.01$ for both scans).

Figure 5b shows results for the low iso-effect doses of hydralazine and prostacyclin (0.1 mg $kg^{-1}$ and 10 ng $kg^{-1}$ $min^{-1}$ respectively). In this case, the two drugs had no

**Figure 5** The effect of high dose hydralazine (1 mg $kg^{-1}$) and prostacyclin (160 ng $kg^{-1}$ $min^{-1}$) (**a**) and low dose hydralazine (0.1 mg $kg^{-1}$) and prostacyclin (10 ng $kg^{-1}$ $min^{-1}$) (**b**) on the peak spectral areas for Pi for the $LBDS_1$ tumour and on the MABP of the host rats. Continuous lines represent Pi levels and broken lines represent MABP as shown in **a**. Filled symbols represent hydralazine and open symbols represent prostacyclin. Values are means ± 1 s.e.m. for 4 rats for hydralazine and 7 rats for prostacyclin in **a** and 3 rats for hydralazine and prostacyclin in **b**. Times are mid-points of spectral collections except that time 0 is the end of the first collection. Filled arrows represent time for injection of hydralazine and start of infusion of prostacyclin. Open arrows represent the time of stopping prostacyclin infusion.

significant effect on tumour Pi (solid lines) ($P > 0.05$ for each value of Pi compared to its pretreatment level, using the Students' $t$ test for paired data) despite a significant reduction in MABP (dashed lines).

Figure 6 shows, more directly, the relationship between MABP and Pi for the two drugs. A single dose of hydralazine is compared with two doses of prostacyclin. Data for 1 mg $kg^{-1}$ hydralazine and 160 ng $kg^{-1}$ $min^{-1}$ prostacyclin are the same as in Figure 5a. Pi only begins to rise when MABP is reduced to below about 65 mmHg. As MABP decreases below 65 mmHg, hydralazine causes a steep increase in Pi. Prostacyclin causes less of an effect on Pi than hydralazine for the same reduction in MABP and reduction of MABP below about 55 mmHg appears to cause no further increase in Pi for this drug.

Figure 7 shows the results for the group of animals in which systemic blood pressure was reduced by controlled bleeding via the tail artery catheter. Measurement of MABP and collection of tumour spectra were carried out simultaneously as described previously. MABP was reduced in stages over a 2 h period down to approximately 45 mmHg (dashed line in Figue 7). This reduction did not have a significant effect on tumour Pi levels (continuous line in Figure 7) ($P > 0.05$ for each value of Pi compared to its pretreatment level, using the Student's $t$ test for paired data).

**Figure 6** The effect of changes in mean arterial blood pressure (MABP) induced by 1.0 mg $kg^{-1}$ hydralazine (filled circles, $n$ = 4), 160 ng $kg^{-1}$ $min^{-1}$ prostacyclin (open squares, $n$ = 7) and 360 ng $kg^{-1}$ $min^{-1}$ prostacyclin (dotted squares, $n$ = 6) on tumour Pi expressed as a % of control values. Each point represents data from a group of animals within a treatment group at different times following administration of drug. Values are means ± 1 s.e.m.

**Figure 7** The effect of controlled bleeding on the peak spectral areas for Pi for the $LBDS_1$ tumour and on the MABP for the host rats. Values are means ± 1 s.e.m. for 4 rats. Times are the mid-points of spectral collections except that time 0 is the end of the first collection. The arrow represents the start of withdrawal of blood.

Tumour pH ($pH_{MRS}$) was calculated for most of the spectra collected. However, sometimes this was not possible due to poor resolution of the PCr or Pi peaks. Figure 8a shows the change in tumour pH for high dose administration of hydralazine (1 mg $kg^{-1}$) and prostacyclin (160 ng $kg^{-1}$ $min^{-1}$). The reduction in pH induced by hydralazine was significant ($P < 0.05$ for each value of pH compared with its pretreatment level, using the Students' $t$ test for paired data) up to 72 min post-injection. The reduction induced by prostacyclin was less than that induced by hydralazine but was still significant throughout the 60 min infusion ($P < 0.05$). It had returned to pre-treatment values after stopping the infusion.

Figure 8b shows the change in tumour pH for low dose administration of hydralazine (0.1 mg $kg^{-1}$) and prostacyclin (10 ng $kg^{-1}$ $min^{-1}$). Tumour pH was significantly reduced from pre-drug levels at 30 min post-injection of hydralazine ($P = 0.015$, Student's $t$ test for paired data) but there were no consistent changes with time and pH at the other time points

**Figure 8** The effect of hydralazine and prostacyclin administration and controlled bleeding on $LBDS_1$ tumour pH. **a** is for high drug doses (1 mg $kg^{-1}$ hydralazine, 160 ng $kg^{-1}$ $min^{-1}$ prostacyclin). **b** is for low drug doses (0.1 mg $kg^{-1}$ hydralazine and 10 ng $kg^{-1}$ $min^{-1}$ prostacyclin). **c** is for controlled bleeding. Times are the mid-points of spectral collections except that time 0 is the end of the first collection. Filled arrows in **a** and **b** represent the time of injection of hydralazine and the start of prostacyclin infusion. The arrow in **c** represents the start of withdrawal of blood. Values are means ± 1 s.e.m., *n* is the number of animals.

was not significantly different from pre-drug pH. Low dose prostacyclin had no significant effect on tumour pH during infusion. The reason for the fall in pH in this group of animals, after stopping the infusion, is not known.

The effect of controlled bleeding on tumour pH is shown in Figure 8c. Tumour pH was reduced significantly for the later time points (>40 min) at which MABP was severely reduced (see Figure 7). The reduction was very similar to that induced by 1 mg $kg^{-1}$ hydralazine for which there was also a comparable reduction in MABP.

The relationship between MABP and tumour Pi and pH is complicated. Summarising the results:

1. High dose hydralazine (1 mg $kg^{-1}$) and high dose prostacyclin (160 ng $kg^{-1}$ $min^{-1}$ and 320 ng $kg^{-1}$ $min^{-1}$) caused substantial falls in MABP. Although the kinetics were different, both drugs were effective in increasing tumour Pi levels and reducing tumour pH.
2. Low dose hydralazine (0.1 mg $kg^{-1}$) and low dose prostacyclin (10 ng $kg^{-1}$ $min^{-1}$) had no significant effect on tumour Pi levels and tumour pH despite a significant reduction in MABP for both drugs.
3. Hydralazine was more effective than prostacyclin in increasing tumour Pi levels and reducing tumour pH for the same *initial* fall in MABP.
4. Reduction in MABP by controlled bleeding was as effective as hydralazine (1 mg $kg^{-1}$) in reducing tumour pH. However, there was no increase in tumour Pi when MABP was reduced by this method.

### Discussion

We have previously shown (Tozer *et al.*, 1989) that the energy metabolism of transplanted rodent tumours is affected by their growth and by treatment with X-rays. These changes are most likely brought about by alterations in tumour blood flow.

Recent studies have shown that i.p. or i.v. administration of hydralazine to mice can decrease PCr, NTP and pH, and increase Pi in several murine tumours growing in different sites (Okunieff *et al.*, 1988; Dunn *et al.*, 1989). Our results are broadly in agreement with these findings. The spectral changes brought about by high dose treatment with hydralazine or prostacyclin are most likely to be largely due to changes in tumour blood flow. A reduction in tumour blood flow has been demonstrated in dogs and in rodents following administration of hydralazine (Voorhees & Babbs, 1982; Horsman *et al.*, 1989). A hydralazine-induced increase in blood flow to normal tissues, without a reduction in tumour blood flow itself, has also been demonstrated, causing a reduction in the ratio of tumour to normal tissue blood flow (Babbs *et al.*, 1982; Chan *et al.*, 1984). The effect of prostacyclin on tumour blood flow has not been studied.

In the present study, tachycardia, following administration of hydralazine and prostacyclin, suggests an increase in cardiac output in an attempt to compensate for the hypotension (cardiac output = heart rate × stroke volume). Vasoactive agents can modify tumour blood flow directly via changes in tumour vascular resistance or indirectly via changes in blood pressure (blood flow through a tissue = arteriovenous pressure difference ÷ vascular resistance). The direct effect is likely to be less than that in normal tissues because tumour blood vessels are generally less well endowed with vascular smooth muscle than normal tissue blood vessels (Warren, 1979). Therefore, any potential drug-induced increase in tumour blood flow resulting from vasodilation of tumour blood vessels and a decrease in vascular resistance was probably masked, in the present experiments, by the hypotensive effect of the two drugs at all doses used. Where there are minimal effects on MABP it is possible to demonstrate an increase in PCr/Pi of animal tumours (Okunieff *et al.*, 1988). At very high doses of the drugs, it is possible that the reduction in tumour blood flow brought about by hypotension is compounded by vascular collapse resulting from a reduction in intravascular pressure to below that of the interstitial pressure (Jain, 1988). This is more likely to occur in tumours than in normal tissues because of their high interstitial pressure (Wiig *et al.*, 1982).

Horsman *et al.* (1989) showed that the radiobiologically hypoxic fraction of a C3H mouse mammary tumour increased and its blood flow decreased following administration of hydralazine. Hypoxia, in the rat fibrosarcoma, resulting from a decrease in tumour blood flow would be expected to cause the observed increase in Pi, decrease in NTP and decrease in PCr of this tumour via the enzyme reactions catalysed by adenylate kinase and creatine kinase. Tumour pH would also be expected to decrease, in the initial stages before depletion of glucose, as a result of an increase in anaerobic glycolysis.

A large reduction in MABP was necessary, for both hydralazine and prostacyclin administration, before any effects on tumour spectra were observed. 'Low dose' hydralazine and prostacyclin caused MABP to fall to approximately

70 mmHg without affecting Pi or pH. 'High dose' drug treatment which produced significant changes in Pi and pH corresponded to a fall in MABP to approximately 55 mmHg. Significant spectral changes were also observed by Okunieff *et al.* (1988) only when MABP was reduced to around 60 mmHg. Such a reduction in MABP is not feasible clinically. Furthermore, the hypotensive effects of hydralazine and prostacyclin in the rat experiments were compounded by the hypotensive effect of general anaesthesia. Equivalent, weight-adjusted drug doses in conscious man would not be expected to be so effective, even if such severe hypotension were feasible. The maximum well-tolerated dose in conscious man for prostacyclin is around 8 mg $kg^{-1}$ $min^{-1}$. This produces a fall in MABP of approximately 15 mmHg and an increase in heart rate of about 20 beats $min^{-1}$ (Lewis & Dollery, 1983).

The whole question of artefacts introduced into spectral measurements by the use of general anaesthesia is an important one. Unfortunately, we have not found a satisfactory method for sufficiently restraining conscious rats for $^{31}$P MRS without causing the animals considerable stress. However, the fact that the results of Okunieff *et al.* (1988), for hydralazine administration to conscious mice, are similar to ours, suggests that anaesthesia is not qualitatively affecting the results.

Despite the reservations regarding effective doses, it is still possible that drugs such as hydralazine and prostacyclin may be clinically useful. Firstly, the sensitivity of changes in energy metabolism, as a marker for changes in tumour blood flow, is not known. Therefore, it is possible that a moderate reduction in tumour blood flow may occur for a moderate fall in MABP. This is suggested by the work of Vaupel (1975). Secondly, an increase in blood flow to the normal tissue surrounding the tumour may occur at moderate reductions in MABP for which tumour blood flow is unchanged. Either of these possibilities would facilitate, for instance, tumour heating for hyperthermia treatments. This would be beneficial, even in the absence of increased thermal sensitivity gained from nutrient deprivation of the tumour itself.

The potential advantage of the short biological half-life of prostacyclin compared with that of hydralazine was outweighed by its apparent reduced effect on spectral parameters for the same initial reduction in MABP. The reason for this differential effect is unclear. Hydralazine acts directly on the vascular smooth muscle of arterioles for its vasodilatory effect although its exact mechanism of action is unknown. Vasodilatation by prostacyclin results from its stimulation of adenylate cyclase which raises intracellular levels of cyclic adenosine monophosphate (Weksler, 1984; Hopkins & Gorman, 1981). This general effect is likely to result in dilatation of all types of blood vessels endowed with smooth muscle.

The net affect of vasodilator on tumour blood flow will be dependent on a balance between its *indirect* effects arising from hypotension caused by vasodilatation in normal tissues and *direct* effects arising from dilatation of the tumour blood vessels themselves. It is therefore possible that prostacyclin has a greater direct dilatory effect on tumour blood vessels than hydralazine. This would tend to counteract the indirect effect of a fall in MABP and maintain blood flow through the tumour. The possibility of direct dilatation of tumour blood vessels depends on (1) their smooth muscle investment, (2) their vascular tone and (3) possession of the relevant receptors. Tumour blood vessels are generally rather poorly endowed with vascular smooth muscle (for review of tumour vascular morphology see Warren (1979)). However, normal blood vessels may be incorporated into the tumour mass during its growth and this will depend upon tumour type (Falk, 1977). Smooth muscle investment also depends upon the size of blood vessels. Falk (1977) found that, for one particular type of fibrosarcoma, both veins and arteries retained a muscular investment until the branching became very fine. Receptor analyses of tumour blood vessels have not been carried out. The balance between indirect and direct effects of vasoactive agents on tumour blood flow has been discussed by Jirtle (1988). The possibility of direct dilatation or constriction of tumour blood vessels requires specific investigation before the therapeutic potential of vasoactive agents can be properly exploited.

Other characteristics of the two drugs could produce a differential effect on tumour blood flow. Prostacyclin is anti-aggregatory for platelets (Pace-Asciak & Gryglewski, 1983). This may be important if platelet aggregation in tumours is significant. Vasodilatation in the normal tissues surrounding the tumour may also be different for the two drugs for the same level of hypotension. We are currently investigating whether the differential effect of hydralazine and prostacyclin in tumour energy metabolism is reflected by a similar differential effect on tumour blood flow.

A direct biochemical effect of hydralazine could also play a role in its effect on tumour energy metabolism. It is known that hydralazine inhibits the action of various enzymes and this may be associated with its ability to chelate metal ions such as $Fe^{2+}$, $Fe^{3+}$ and $Cu^{2+}$ (Gross, 1977). Hydralazine is metabolised in the body by acetylation via acetyl coenzyme a which is an ATP requiring process (Douglass *et al.*, 1957). It is also known (Gross, 1977) that sublethal doses of hydralazine can reduce levels of high energy phosphates in the brain and depress oxygen uptake in preparations of brain, liver and kidney of rats.

Depite the relationship demonstrated between drug-induced systemic hypotension and tumour Pi (Figure 6), systemic hypotension is not, on its own, a good indication of changes in the tumour micro-environment. This is illustrated by the spectral changes observed for rats whose MABP was reduced by controlled bleeding. Reduction of MABP to levels comparable with those induced by hydralazine was obtained by this method. However, although tumour pH was also reduced comparably, tumour Pi remained unchanged. This result is difficult to explain. Vaupel (1975) has investigated the effect of controlled bleeding on MABP and blood flow in the DS-carcino-sarcoma implanted into rat kidneys. He showed that blood flow was linearly related to MABP within the range of 40–135 mmHg. If this is the case for the subcutaneous fibrosarcoma in the current experiments then one would expect Pi to be significantly affected. Catecholamine release, due to blood loss, would tend to increase glycolysis and this may be sufficient to maintain high energy phosphate levels under these conditions. An increase in lactate levels would then explain the decrease in pH. Another possiblity is that venous pressure will be reduced following controlled bleeding and this may, in turn, reduce tumour interstitial pressure. Such a reduction could maintain tumour blood flow despite a fall in arterial blood pressure. Obviously the relationship between MABP, tumour phosphate levels and tumour pH is very complicated and requires further investigation.

In conclusion, $^{31}$P MRS was useful for monitoring the effects of vasoactive drugs on tumour energy metabolism. Vasoactive drugs such as hydralazine and prostacyclin, which appear to affect tumour energy metabolism primarily via their vasodilatory effects on normal tissues, which reduces systemic blood pressure, may be ineffective in altering tumour energy metabolism clinically because of the severe systemic hypotension involved. The differential between the effects of hydralazine, prostacyclin and controlled bleeding on tumour energy metabolism, for the same degree of systemic hypotension, suggests further studies on the direct versus indirect effects of hydralazine on tumour energy metabolism. This would be desirable for a systematic approach to finding methods for manipulating tumour blood flow for optimisation of therapy.

We would like to thank the Wellcome Foundation for our supply of prostacyclin. R.J.M. and J.R.G. thank the Cancer Research Campaign for financial support. We also thank Dr J. Ritter, Dr C. Newman, Professor B.F. Robinson and Dr C. Wilson for useful scientific discussion. We thank Mr T. Jenkinson and his staff for care of the animals.

### References

ARMSTRONG, J.M., CHAPPLE, D., DUSTING, G.J., HUGHES, R., MONCADA, S. & VANE, J.R. (1977). Cardiovascular actions of prostacyclin ($PGI_2$) in chloralose anaesthetized dogs. *Br. J. Pharmacol.*, **61**, 136P.

BABBS, C.F., DEWITT, D.P., VOORHEES, W.D., MCCAW, J.S. & CHAN, R.C. (1982). Theoretical feasibility of vasodilator-enhanced local tumor heating. *Eur. J. Cancer Clin. Oncol.*, **18**, 1137.

BROWN, J.M. (1987). Exploitation of bioreductive agents with vasoactive drugs. In *Proc. 8th Int. Congress of Radiation Research*, p. 737. Taylor & Francis: London.

CHAN, R.C., BABBS, C.F., VETTER, R.J. & LAMAR, C.H. (1984). Abnormal response of tumor vasculature to vasoactive drugs. *J. Natl Cancer Inst.*, **72**, 145.

CHAPLIN, D.J. & ACKER, B. (1987). The effect of hydralazine on the tumour cytotoxicity of the hypoxic cell cytotoxin RSU-1069: evidence for therapeutic gain. *Int. J. Radiat. Oncol. Biol. Phys.*, **13**, 579.

CHIAVARELLI, M., MONCADA, S. & MULLANE, K.M. (1982). Prostacyclin can either increase or decrease heart rate depending on the basal state. *Br. J. Pharmacol.*, **75**, 243.

DOUGLASS, C.D., DILLAHA, M.D., DILLAHA, J. & KOUNTZ, S.L. (1957). Inhibition of biological acetylation by 1-hydrazinophthalazine. *J. Lab. Clin. Med.*, **49**, 561.

DUNN, J.F., FROSTICK, S., ADAMS, G.E. & 4 others (1989). Induction of tumour hypoxia by a vasoactive agent. A combined NMR and radiobiological study. *FEBS Letts*, **249**, 343.

FALK, P. (1977). The angio-architecture of rat tumours. *Bibl. Anat.*, **15**, 245.

GROSS, F. (1977). Drugs acting on arteriolar smooth muscle (vasodilator drugs). In *Antihypertensive Agents*, Gross, F. (ed.) p. 37. Springer-Verlag: Berlin.

HAHN, G.M. (1974). Metabolic aspects of the role of hyperthermia in mammalian cell activation and their possible relevance to cancer treatment. *Cancer Res.*, **34**, 311.

HAHN, G.M. & SHIU, E.C. (1986). Adaption to low pH modifies thermal and thermochemical responses of mammalian cells. *Int. J. Hypertherm.*, **2**, 379.

HOPKINS, N.K. & GORMAN, R.R. (1981). Regulation of endothelial cell cycle nucleotide metabolism by prostacyclin. *J. Clin. Invest.*, **67**, 540.

HORSMAN, M.R., CHRISTENSEN, K.L. & OVERGAARD, J. (1989). Hydralazine-induced enhancement of hyperthermic damage in a C3H mammary carcinoma *in vivo*. *Int. J. Hypertherm.*, **5**, 123.

JAIN, R.K. (1988). Determinants of tumour blood flow: a review. *Cancer Res.*, **48**, 2641.

JAIN, R.K. & WARD-HARTLEY, K. (1984). Tumour blood flow – characterization, modifications and role in hyperthermia. *IEEE Transactions of Sonics and Ultrasonics*, **SU-31**, 504.

JIRTLE, R.L. (1988). Chemical modification of tumour blood flow. *Int. J. Hypertherm.*, **4**, 355.

LEWIS, P.J. & DOLLERY, C.T. (1983). Clinical pharmacology and potential of prostacyclin. *Br. Med. Bull.*, **39**, 281.

MONCADA, S., GRYGLEWSKI, R.J., BUNTING, S. & VANE, J.R. (1976). An enzyme isolated from arteries transforms prostaglandin endoperoxides to an unstable substance that inhibits platelet aggregation. *Nature*, **263**, 663.

O'GRADY, J., WARRINGTON, S., MOTI, M.J. & 5 others (1980). Effects of intravenous infusion of prostacylin ($PGI_2$) in man. *Prostaglandins*, **19**, 319.

OKUNIEFF, P., KALLINOWSKI, F., VAUPEL, P. & NEURINGER, L.J. (1988). Effects of hydralazine-induced vasodilation on the energy metabolism of murine tumors studied by *in vivo* 31P-nuclear magnetic resonance spectroscopy. *J. Natl Cancer Inst.*, **80**, 745.

ORCHARD, M.A. & ROBINSON, C. (1981). Stability of prostacyclin in human plasma and whole blood. Studies on the protective effect of albumin. *Thromb. Haemost.*, **46**, 645.

OVERGAARD, J. & BICHEL, P. (1977). The influence of hypoxia and acidity on the hyperthermic response of malignant cells *in vitro*. *Radiology*, **123**, 511.

OVERGAARD, J. & NIELSEN, O.S. (1980). The role of tissue environmental factors on the kinetics and morphology of tumour cells exposed to hyperthermia. *Ann. NY Acad. Sci.*, **335**, 254.

PACE-ASCIAK, C. & GRYGLEWSKI, R. (1983). The prostacyclins. In *Prostaglandins and Related Substances*, Pace-Asciak, C. & Granstrom, E. (eds), p. 95. Elsevier: Amsterdam.

PRICHARD, J.W., ALGER, J.R., BEHAR, K.L., PETROFF, O.A.C. & SHULMAN, R.A. (1983). Cerebral metabolic studies *in vivo* by $^{31}$P-NMR. *Proc. Natl Acad. Sci. USA*, **80**, 2748.

SHEPHERD, A.M.M., LUDDEN, T.M., MCNAY, J.L. & LING, M.-S. (1980). Hydralazine kinetics after single and repeated oral doses. *Clin. Pharmacol. Ther.*, **28**, 804.

STRATFORD, I.J., GODDEN, J., HOWELLS, N., EMBLING, P. & ADAMS, G.E. (1987). Manipulation of tumour oxygenation by hydralazine increases the potency of bioreductive radiosensitizers and enhances the effect of melphalan in experimental tumours. In *Proc. 8th Int. Congress of Radiation Research*, p. 737. Taylor & Francis: London.

TOZER, G.M., BHUJWALLA, Z.M., GRIFFITHS, J.R. & MAXWELL, R.J. (1989). Phosphorus-31 magnetic resonance spectroscopy and blood perfusion of the RIF-1 tumor following X-irradiation. *Int. J. Radiation Oncology Biol. Phys.*, **16**, 155.

TOZER, G.M. & MORRIS, C.M. (1990). Blood flow and blood volume in a transplanted rat fibrosarcoma: comparison with various normal tissues. *Radiother. Oncol.*, **17**, 153.

VAUPEL, P. (1975). Interrelationship between mean arterial blood pressure, blood flow and vascular resistance in solid tumor tissue of DS-carcinosarcoma. *Experentia*, **31**, 587.

VOORHEES, W.D. & BABBS, C.F. (1982). Hydralazine-enhanced selective heating of transmissible venereal tumor implants in dogs. *Eur. J. Cancer Clin. Oncol.*, **18**, 1027.

WARREN, B.A. (1979). The vascular morphology of tumors. In *Tumor Blood Circulation: Angiogenesis, Vascular Morphology and Blood Flow of Experimental and Human Tumors*, Peterson, H.-I. (ed.) p. 1. CRC Press: Boca Raton, FL.

WEKSLER, B.B. (1984). Prostaglandins and vascular function. *Circulation*, **70**, III-63.

WIIG, H., TVEIT, E., HULTBORN, R., REED, R.K. & WEISS, L. (1982). Interstitial fluid pressure in DMBA-induced rat mammary tumours. *Scand. J. Clin. Lab. Invest.*, **42**, 159.